# Structure-Dependent Activity of Plant-Derived Sweeteners

**DOI:** 10.3390/molecules25081946

**Published:** 2020-04-22

**Authors:** Serhat Sezai Ҫiçek

**Affiliations:** Department of Pharmaceutical Biology, Kiel University, Gutenbergstraße 76, 24118 Kiel, Germany; scicek@pharmazie.uni-kiel.de; Tel.: +49-431-880-1077

**Keywords:** natural product, plant origin, non-caloric sweeteners, mogrosides, stevia glycosides, phyllodulcin

## Abstract

Human sensation for sweet tastes and the thus resulting over-consumption of sugar in recent decades has led to an increasing number of people suffering from caries, diabetes, and obesity. Therefore, a demand for sugar substitutes has arisen, which increasingly has turned towards natural sweeteners over the last 20 years. In the same period, thanks to advances in bioinformatics and structural biology, understanding of the sweet taste receptor and its different binding sites has made significant progress, thus explaining the various chemical structures found for sweet tasting molecules. The present review summarizes the data on natural sweeteners and their most important (semi-synthetic) derivatives until the end of 2019 and discusses their structure–activity relationships, with an emphasis on small-molecule high-intensity sweeteners.

## 1. Introduction

**Figure 1:**
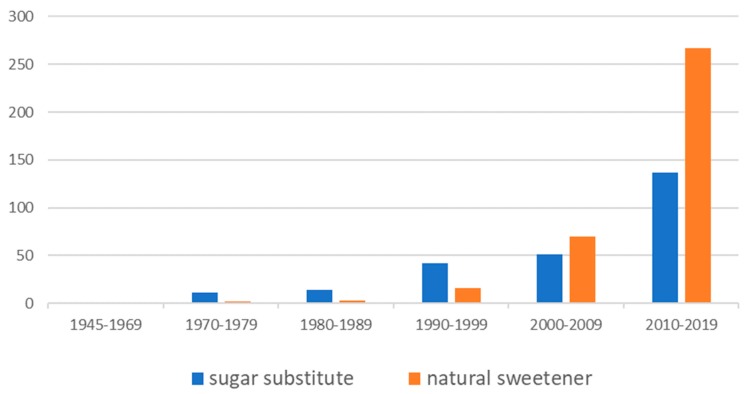
Number of publications with the topic “sugar substitute” and “natural sweetener” according to the Web of Science citation indexing service (core collection) from 1945 to 2019.

Taste—or the gustatory system—is the essential evolutionary tool to evaluate the composition of foods before ingestion [1,2]. The five taste qualities generally considered to be basic tastes are sweet, sour, salty, bitter, and umami, the latter being typically elicited by L-glutamate [2,3]. Each of these five taste qualities plays an important role, either indicating high electrolyte concentrations (salty), spoiled food (sour), and poisonous plant metabolites (bitter), or giving nutritional information, such as the abundance of carbohydrates (sweet) or proteins (umami) [3,4]. Sweet and umami are the main attractive taste modalities and therefore called the two palatable tastes [5,6]. In particular, the sweet taste has a great relevance, as most people respond positively to the sensation of sweetness [7]. The propensity to sweet foods and the thus resulting over-consumption of sugar in industrial countries has led to a significant number of people suffering from caries, diabetes, and hyperlipidemia [7,8,9]. Consequently, in the last four decades a market for non-caloric sweeteners and dietary products has evolved, addressing the needs of more than a billion people [10]. Additionally, more and more consumers express their interest for natural ingredients, leading to an increasing demand for natural non-caloric sweeteners [10]. This demand is reflected by scientific publications, which show both an increase in papers dealing with sugar substitutes and a significant rise in publications on natural sweeteners (Figure 1).

Over the years, numerous compounds have been identified with sweetening properties, either by phytochemical approaches or organic synthesis [11,12,13]. In many cases, natural compounds or their glucophore were taken as lead compound, such as the isovanillyl group of (+)-phyllodulcin, which is the sweet principle of amacha (sweet tea), a regional specialty in Japan [14]. (+)-Phyllodulcin and its analogs were furthermore studied to understand the structure–activity relationships of the sweetening effect. The first successful model was established by Schallenberger and Acree, who hypothesized that a hydrogen bond donor and a hydrogen bond acceptor were interacting with a complementary pair of hydrogen bond donor and acceptor on the receptor, and that this feature would be the crucial interaction for binding [15]. Other models followed, applying either planar [16] or three-dimensional geometries, the latter deriving from newly found guanidine-based sweeteners [17]. However, due to the ongoing discovery of natural sweeteners with distinct chemical structures, the idea of a general model to predict and quantify the sweetness of compounds was abandoned, now assuming that different classes of sweet molecules might interact with different receptor types [18]. Still, the model of Schallenberger and Acree was furthermore used to describe the linkage between sweetness and structure, as demonstrated in recent rotational studies on sugars and artificial sweeteners combining laser ablation with Fourier-transform microwave spectroscopy [19,20,21].

The identification of the taste 1 receptor family, comprising three taste receptors (TAS1R1–3), revealed that indeed only one receptor type is responsible for the sweet taste, which is a heterodimer of TAS1R2 and TAS1R3 [22,23]. Furthermore, the taste for umami was attributed to the TAS1R1/TAS1R3 heterodimer, thus showing that both taste receptors share a common subunit [5,23]. Same as other class C G Protein-coupled receptors (GPCRs), the sweet taste receptor is composed of a cytoplasmic region, a region constituted by seven transmembrane helices, and a large extracellular region [4,22]. In contrast to some other GPCRs, such as metobotropic glutamate receptors or γ-aminobutyric acid type B receptors, which function as homo- and heterodimers, TAS1 receptors are obligatory heterodimers [2]. The extracellular domain contains a Venus fly trap domain (VFD) and a cysteine-rich domain (CRD), with nine cysteine residues forming four disulfide bonds and a fifth disulfide bond with the VFD. Even though several GPCRs have already been crystallized, a crystal structure for the sweet taste receptor is still missing [9,22]. However, the good sequence alignment with metabotropic glutamate receptors 1 and 5 allowed a homology 3D-model of the sweet taste receptor and a detailed discussion of the different binding sites [9]. The orthosteric binding pockets both have a volume of about 4900 Å³ in their open form and thus allow small as well as large sweeteners to bind to the receptor. Additionally, both cavities are hydrophilic, with 45% (TAS1R2) and 50% (TAS1R3) surface area being accessible to polar molecules. Furthermore, each subunit contains a transmembrane domain (TMD) binding pocket, with a volume of 210 Å³ for TAS1R2 and 270 Å³ for TAS1R3, respectively. With the TAS1R3 TMD binding pocket also being present in the umami receptor, ligands such as the sweetener cyclamate or the sweetness inhibitor lactisol have also been found to enhance or inhibit the taste of glutamate [24]. Both binding sites allow allosteric modulation of small molecules, while another allosteric binding site is in the CRD and accessible for macromolecules, such as the sweet-tasting proteins brazzein, monellin or thaumatin [25]. The orthosteric binding sites in the VFD of both subunits can bind ligands, which is the case for e.g., sucrose, but most sweeteners mainly interact with the binding site in the VFD of TAS1R2 [18]. Recent findings, furthermore, suggest that the binding site in the VFD of TAS1R3 plays an auxiliary role, showing less discriminating recognition characterized by loosely bound amino acids [6]. Interestingly, the signal transduction (G protein-coupling) is also carried out by the TMD of the TAS1R3 subunit, after having been transmitted from the VFD of TAS1R2, via the VFD of TAS1R3 and the CRD of TAS1R3, respectively [26].

## 2. Method

Literature search was carried out using the Web of Science citation indexing service and the term “sweetener” in combination with the words “natural product”, “naturally occurring” or “plant-derived” resulting in 436 publications. Additionally, the terms “natural” and “sweeteners” were combined, and the results were reduced to the field of plant science and multidisciplinary chemistry, giving 155 hits. The publications were reviewed by title, abstract, and text, and reduced. Additionally, studies from other review articles were collected, which were not found by database search. Chemical structures were divided into compound classes (carbohydrates, amino acids, phenols, and terpenes) and, depending on the number compounds, into subclasses, thereby defining the structure of this review. Compound names (mostly trivial names) and configurations were taken “as is” from the original publications and species names of the natural sources used in the studies were checked using “The Plant List” [27].

The data discussed in this review is additionally summarized in two tables, giving an overview of natural high-intensity sweeteners (Table 1) and those which were chemically modified (Table 2). Here, compound number, name, and source are given, as well as relative sweetness (RS) values and the concentration of the sucrose solution used for comparison (if available). Furthermore, concomitant bitter tastes (or aftertastes) of the compounds are indicated and divided into slightly bitter (+), bitter (++) and very bitter (+++). (–) means that the compound was reported to exhibit no bitter taste, whereas blank fields indicate the lack of respective data.

## 3. Plant-Derived Sweeteners

### 3.1. Carbohydrates

#### 3.1.1. Sugars

**Figure 2:**
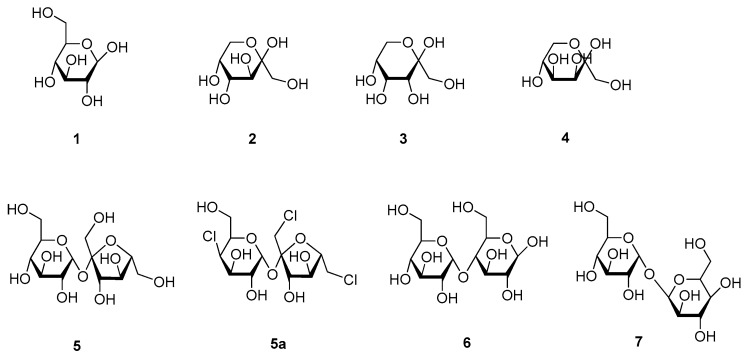
Chemical structures of sweet-tasting sugars and derivatives.

Carbohydrates are an important dietary source, with mono- and disaccharides providing fast energy. However, not only their content on calories made this compounds valuable to humans, also their sweet taste let them collect and culture different forms of this nutrients for thousands of years [11]. First historical records were found for honey, which consist largely of D-glucose (**1**) and D-fructose (**2**), the two monomers of the sucrose disaccharide (**5**) (Figure 2) [11,28]. D-glucose (**1**) only has RS of 0.5 to 0.8 compared to sucrose (**5**), while the RS of D-fructose (**2**) is ranging from 1.1 to 1.7, making it the sweetest naturally occurring sugar [11,29]. The mixture of the two monosaccharides, which is called “invert sugar”, is about 0.8 times as sweet as sucrose (**5**), while honey itself is sweeter than sugar, because of higher content of D-fructose (**2**) (32 to 38%) compared to D-glucose (**1**) (28 to 31%) [28,30]. Apart from **1** and **2**, additional monosaccharides are known to exhibit a sweet taste, such as D-allulose (**3**), which is also known as D-psicose, and D-tagatose (**4**) [31,32]. Showing RS values of 0.7 and 0.9, respectively, and being generally recognized as safe (GRAS) by the Food and Drug Administration, both compounds can be used as sugar substitutes.

Sucrose (**5**), also known as saccharose, or table sugar, has been used to flavor food for centuries; being first harvested from sugarcane it is meanwhile also obtained from the sugar beet [11]. Despite its many advantages, such as its easy availability from natural sources or its use as preservative, consumption of high amounts of sucrose (**5**) can cause health problems, such as caries, obesity, or diabetes, and therefore led to the search for alternative sweeteners, either from plant origin or by (semi)synthetic synthesis [11,32,33]. One of the most used sweeteners is sucralose (**5a**), which is obtained by chlorination of sucrose (**5**) and exhibits a similar taste, but with an about 600 times higher intensity [32]. Though sucralose (**5a**) underwent extensive safety evaluations, there have been some concerns that the compound leads to a decrease in beneficial gut bacteria and to thus provoke adverse effect in the gastrointestinal tract [34,35]. Other sweet-tasting disaccharides used in food industry are maltose (**6**) and trehalose (**7**), though both compounds show less than half of the sweetness of sucrose (**5**) [27,36,37].

Regarding receptor interaction, glucose (**1**), sucrose (**5**), and sucralose (**5a**) in contrast to many other sweeteners, are known to bind to the orthosteric binding sites of both subunits [38]. Due to the structural similarities of the other saccharides, their relatively similar intensities, and the fact that the orthosteric binding sites are accessible to polar molecules, it can be assumed that these molecules show similar binding [9]. However, the reason for the much higher intensities of sucralose (**5a**) and other chlorinated sucrose derivatives seems to derive from the much higher affinity to the TAS1R3 subunit than its counterpart sucrose (**5**), while at the same time showing comparable affinities to the TAS1R2 binding site [38]. Another reason is that the orthosteric binding sites show only 45% and 50% polar surface, respectively, meaning that additional lipophilic groups might enhance the sweetening effect and contribute to the much higher intensities of sucralose (**5a**) and other chlorinated sucrose derivatives [9,39]. Here, in particular the chlorine atom at the C1′ of the furanose moiety showed favorable hydrophobic interactions [40].

A study on the conformational behavior of ketohexoses by new spectroscopic techniques and laser ablation methods, found that the most abundant sweet-tasting conformers all show the same intramolecular H-bond network, with the anomeric hydroxy-group always in axial orientation and the primary hydroxy-group pointing towards the cyclically bound oxygen [19]. Apart from the same conformational signature, these two hydrogen atoms were found to be exclusively responsible for the sweet taste. Therefore, the primary hydroxy-group is acting as proton donor and the axial hydroxy-group as proton acceptor, thus corroborating the theory of Schallenberger and Acree [15].

#### 3.1.2. Sugar Alcohols

**Figure 3:**
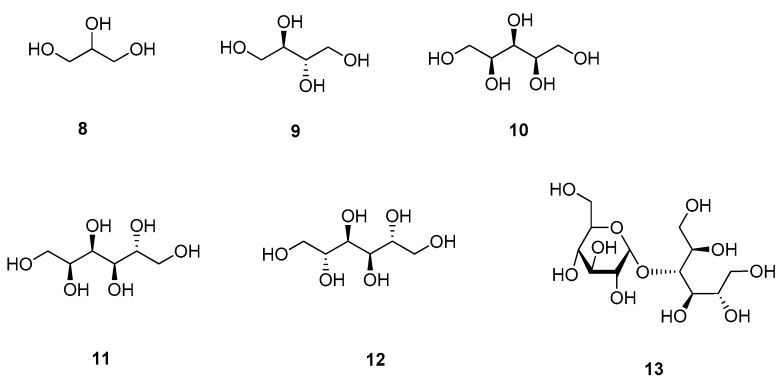
Chemical structures of sweet-tasting sugar alcohols.

Apart from mono- and disaccharides, also their reduced forms, the so-called sugar alcohols or polyols, can exhibit a sweet taste and are used as alternative sweeteners [10,11,32,33]. The smallest polyol is glycerol (**8**), though the compound is not used because of its sweetening effect (Figure 3) [7]. Erythritol (**9**) instead, is widely used in food industry, because of its high stability, its clean sweet taste and the lack of unpleasant aftertastes [10,33]. Its sweetness is reported to be about 0.6 to 0.7 times that of sucrose (**5**), but combination with other sweeteners can increase its sweetness by up to 30%. Other commonly used sugar alcohols are mannitol (**10**), sorbitol (**11**) and xylitol (**12**) [11,33]. While mannitol (**10**) and sorbitol (**11**) show RS values of 0.5 to 0.7, xylitol has a sweetness equal to that of sucrose (**5**) and thus is the sweetest sugar alcohol known. Maltitol (**13**) is a twelve-carbon sugar alcohol, which is also occurring in nature but same as other sugar alcohols is usually obtained by catalytic hydrogenation [11]. Its RS value is 0.9 and thus higher than that of most other sugar alcohols, but it also has a considerably higher glycemic index (35) than its congeners [11,33].

Sugar alcohols differ from their saccharidic counterparts by only two protons, which results from hydrogenation of one carbonyl group. Therefore, these compounds can be expected to share the same binding site, which is also reflected by their intensities. However, the use of sugar alcohols does not so much result from their intense sweetness, but from their similar taste to sucrose (**5**) and their additional values, e.g., protection of tooth decay in chewing gums [11]. However, when it comes to their use as substitutes in dietary foods, their caloric values as well as their glycemic index must be taken into consideration. Apart from that, most sugar alcohols show low absorption in the upper gastrointestinal tract and are subsequently fermented by colon bacteria, leading to flatulence and digestion problems [33].

Same as the abovementioned ketohexoses, sorbitol (**11**) and the linkage between its sweetness and structure were investigated in a rotational study using laser ablation and Fourier-transform microwave spectroscopy [20]. Interestingly, in all three observed conformers the hydroxy-groups at positions 2 to 6 were involved in a circular intramolecular hydrogen bond network, whereas the hydroxy-group at position 1 showed no interaction with any part of the molecule. Regarding the model of Schallenberger and Acree and the abovementioned study on sweet-tasting ketohexoses, the hydroxy-group at position 1 again fulfils the role of the proton donor, while the hydroxy-group at position 2 serves as proton acceptor, the latter being accomplished by the intramolecular hydrogen bonds and the thus resulting disposition of the oxygen atom [15,19,20].

### 3.2. Amino Acids

**Figure 4:**
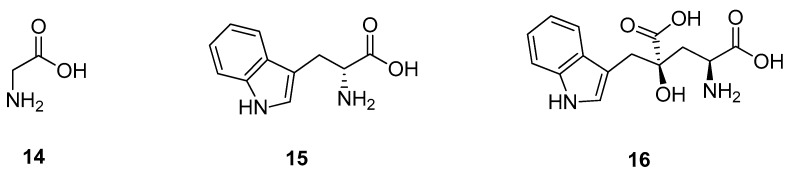
Chemical structures of sweet-tasting amino acids and monatin.

Most naturally occurring amino acids are tasteless or bitter, except alanine and glycine (**14**) (Figure 4) [41]. In fact, the word glycine even derives from the Greek word of sweet [7]. Of the bitter amino acids, interestingly, their unnatural D-forms show a sweet taste, such as d-phenylalanine, d-tyrosine, or d-tryptophan (**15**) [41]. Additionally, there are several sweet synthetic or natural peptides, such as the artificial sweetener aspartame or monatin (**16**), which is the first natural high potency sweetener to be presented in this review [42,43,44]. Monatin (**16**) was isolated from the roots of *Schlerochiton ilicifolius*, a South African species of the Acanthaceae family in 1992. Evaluation of the compound sweetening effect with 5 and 10% (*w*/*v*) sucrose solutions gave RS values of 1400 and 1200, respectively, and thus a promising new natural sweetener. Even more so, as in subsequent studies three out of four diastereomers were found to be sweet and chemo-enzymatic approaches for the synthesis of the more potent enantiomers were established [45,46].

D-tryptophan (**15**) was found to bind to the same binding site as aspartame and thus exclusively to the VFD of the TAS1R2 subunit [26,40]. Monatin (**16**) is a dipeptide consisting of tryptophan and glycine, which is also part of the synthetic dipeptide aspartame, and therefore binding to the same binding pocket must be assumed.

### 3.3. Phenols

#### 3.3.1. Phyllodulcin and Derivatives

**Figure 5:**
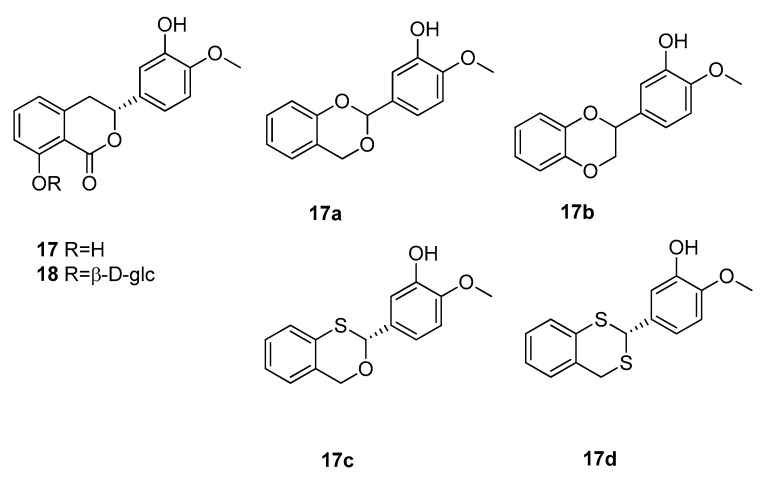
Chemical structures of sweet dihydroisocoumarins and derivatives.

The next high potency sweetener to be discussed is (+)-phyllodulcin (**17**) (Figure 5), which is the sweetening principle of amacha, a traditional Japanese preparation [14]. Amacha means “sweet tea” and is obtained by fermenting leaves of *Hydrangea macrophylla* (Hydrangeaceae), which leads to the enzymatic hydrolyses of (+)-phyllodulcin-8-*O*-β-d-glucoside (**18**) into the much sweeter aglycone **17**. Having already been isolated in 1929, (+)-phyllodulcin (**17**) served as lead compound for the development of new sweeteners and for models to describe their structure–activity relationships [47]. These studies focused on the isovanillyl group, which is responsible for the sweetening effect and resulted in the synthesis of several derivatives [48]. First attempts revealed two dioxane derivatives (**17a** and **17b**), of which compound **17a** showed an RS value of 3000 but low stability in aqueous media [13]. Compound **17b** instead remained stable, but the sweetening effect decreased to “only” 450 times that of glucose. However, subsequent synthesis of oxathiane (**17c**) and dithiane (**17d**) derivatives with RS values of 18,000 and 20,000, for the sweet (*R*)-enantiomer.

Neohesperidin dihydrochalcone, a commonly used semi-synthetic sweetener, was found to interact with the TMD binding pocket in the TAS1R3 subunit and thus on the same site as cyclamate and the sweetness inhibitor lactisol [49]. Because neohesperidin dihydrochalcone belongs to the class of isovanillyl derivatives, also (+)-phyllodulcin (**17**) and its analogs are expected to bind to this site. The allosteric receptor modulation via this binding site furthermore explains the extremely high RS values of some of the synthesized compounds.

#### 3.3.2. Flavanonols

**Figure 6:**
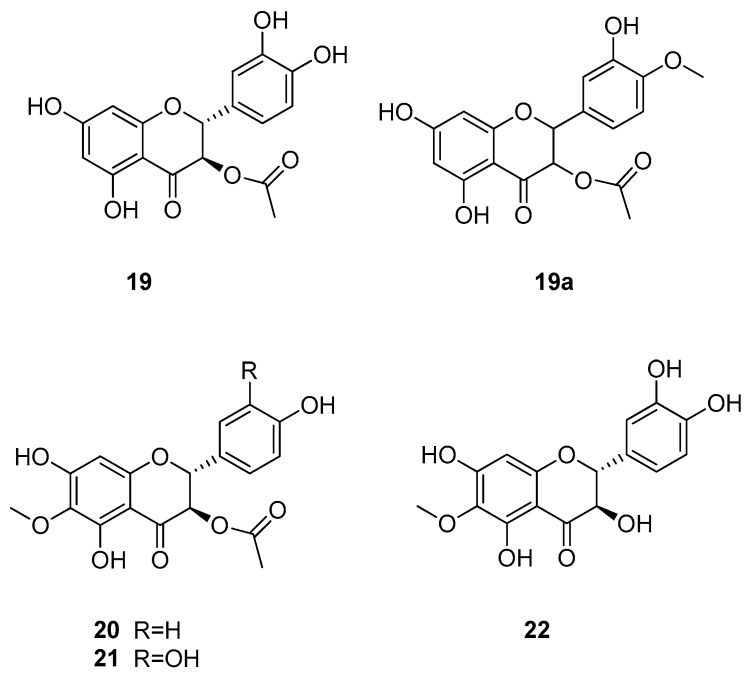
Chemical structures of sweet-tasting flavanonols.

Another compound class which yielded sweet-tasting constituents is the class of flavanonols, also referred to as diyhdroflavonols (Figure 6). Of this compound class so far, four natural sweet-tasting molecules have been isolated. (+)-Dihydroquercetin acetate (**19**) was isolated from the young shoots of *Tessaria dodoneifolia* (Asteraceae), which was meanwhile classified into the genus *Pluchea* [50]. The compound showed an RS value of 80 compared to a 2% (*w*/*v*) sucrose solution. However, a synthetic analog (**19a**) showing a 4′-methoxy-group and thus an isovanillyl moiety, exhibited a RS of 400. Because only the racemic form was synthesized it can be assumed that the (+)-enantiomer shows twice the intensity. Further flavanonols (**20–22**) have been isolated from *Hymenoxys turneri* (Asteraceae), which now belongs to the genus *Tetraneuris*, but their RS values were only in the range of 15 to 25 [51].

#### 3.3.3. Dihydrochalcones

**Figure 7:**
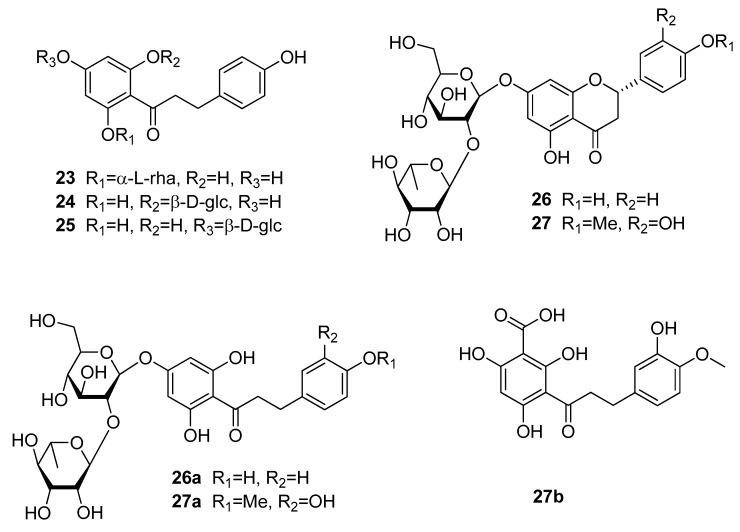
Chemical structures of sweet-tasting dihydrochalcones and their precursors.

The class of dihydrochalcones contains three semi-synthetic high potent sweeteners (**26a**, **27a**, and **27b**), though their precursor molecules are ranging from slightly sweet (**23–25**) to even bitter molecules (**26** and **27**) (Figure 7). Compounds **23** to **25** are naturally occurring dihydrochalcones, so-called phloretin derivatives. The first of these compounds, glycyphyllin (**23**) was isolated from *Smilax glycyphylla* (Smilaccaceae), which is meanwhile named *Smilax leucophylla*, and was reported to have a bittersweet taste, similar to that of licorice [11]. Phlorizin (**24**) was obtained from *Symplocos lancifolia* (Symplocaceae), while trilobatin (**25**) was isolated form *Symplocos microcalyx*, a species with an unresolved taxonomic status [52,53].

The sweetening potential of naringenin dihydrochalcone (**26a**) and especially neohesperidin dihydrochalcone (**27a**) was already detected in 1969, when the respective flavanones (**26** and **27**) were treated with hot alkali [54]. While compound **26a** shows an RS value of 300, neohesperidin dihydrochalcone (**27a**) shows a relative sweetness of 1000 compared to a 5% (*w*/*v*) sucrose solution. The higher intensity of compound **27a** derives from the isovanillyl feature, which is missing in naringenin dihydrochalcone (**26a**). Though neohesperidin dihydrochalcone is used as sweetener in a wide range of foods and beverages it has some limitations, such as a slow onset and a lingering aftertaste [12,55,56]. To improve these temporal deficits different modification of neohesperidin have been carried out, with one compound, 3′-carboxyhesperetin dihydrochalcone (**27b**), even showing a higher RS 3400 compared to a 6% sucrose solution [57]. However, the characteristic lingering aftertaste was not improved.

Same as above, the isovanillyl feature of compounds **27a** and **27b** was responsible for the much higher sweetening effect compared to compounds lacking this feature (**23–26a**). Compared to the previously discussed phenolic compound classes, the dihydrochalcone type seems to stimulate the allosteric binding pocket to a much higher extent than other plant-derived phenols.

#### 3.3.4. Condensed Phenols

**Figure 8:**
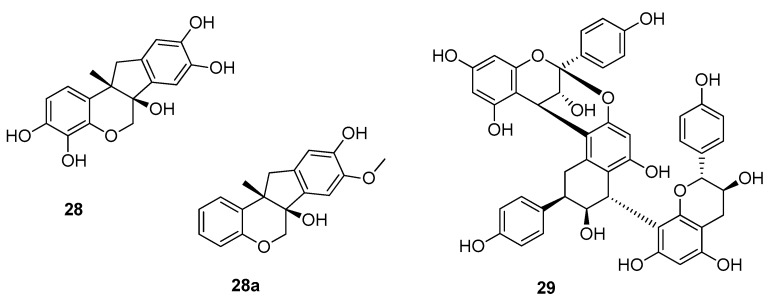
Chemical structures of sweet-tasting condensed phenols.

The last class of phenolic compounds to be discussed consists of two condensed phenols (**28** and **29**) and one synthetic analog (**28a**) (Figure 8). The first compound is (+)-haematoxylin (**28**), a constituent of *Haematoxylum campechianum* (Fabaceae) and shows an RS value of 120 compared to a 3% (*w*/*v*) sucrose solution [58]. To improve the sweetening effect a synthetic analog was prepared, which displayed an isovanillyl feature on one of the two aromatic rings. However, the relative sweetness did not alter significantly, now showing an RS value of 50 for the racemate.

The second condensed phenol is a proanthocyanidine isolated from the Indonesian medicinal plant *Selliguea feei* (Polypodiaceae) [59]. The bittersweet rhizomes, which are used for the treatment of rheumatism and as tonic, yielded selligueain A (**29**), which showed a RS of 35, when compared to a 2% (*w*/*v*) sucrose solution.

Structure–activity relationships of these three compounds are difficult. Due to their phenolic nature and it must be assumed that they share the same binding pocket as the previously discussed phenols. However, the fact that compound **28a** did not show significantly higher RS values than its congener as well as the size of compound **29** somehow contradict this theory. On the contrary, the relatively low intensities observed for these molecules in comparison to e.g., diyhdrochalcones could hint towards the same binding site but much lower efficacies.

### 3.4. Monoterpenes

**Figure 9:**
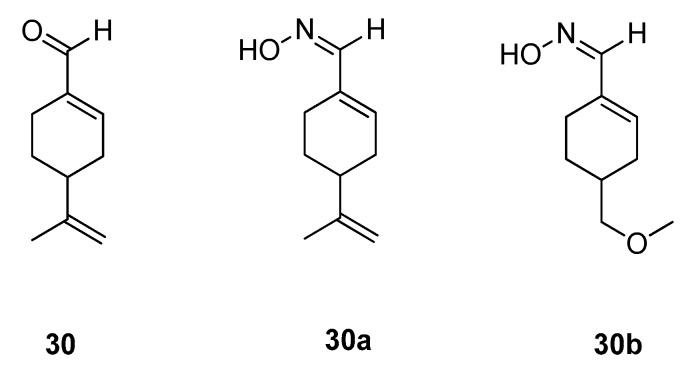
Chemical structures of sweet-tasting monoterpenes and derivatives.

The class of monoterpenes, so far, only yielded one natural sweetener, which is perillaldehyde (**30**) (Figure 9). The compound is the sweet principle of perilla oil, which is obtained by distillation of *Perilla frutescens* (Lamiaceae) [11]. The compound itself, same as the oil, only shows a slightly sweet taste, but the respective *syn*-oxime, perillartine (**30a**) possesses a RS of about 370 to that of sucrose (**5**) [11,60,61]. However, perillartine (**30a**) has an appreciable bitterness and a low water solubility, thus restricting its use for many applications [61]. Further studies focusing on perillartine analogs yielded compound **30b**, which showed an improved RS of 450 times to that of sucrose (**5**) coupled with superior water solubility [61].

Apart from the TMD binding pocket of the TAS1R3 subunit, also the TAS1R2 subunit has an allosteric binding site [9,26]. The latter is said to have a volume of about 210 Å³ and, moreover, has a surface area of 38% accessible to polar molecules [9]. Thus, only very small and less hydrophilic molecules are known to bind to this region, such as (+)-perillartin (**30a**) and its analogs.

### 3.5. Sesquiterpenes

**Figure 10:**
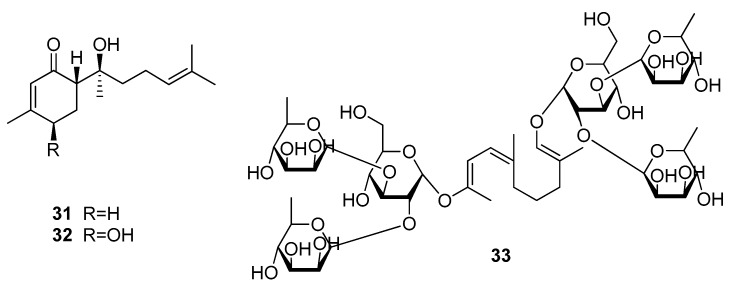
Chemical structures of sweet-tasting sesquiterpenes.

Another two sweet-tasting volatile oil constituents belong to the class of sesquiterpenes and are named (+)-hernandulcin (**31**) and (+)-4β-hydroxyhernandulcin (**32**) (Figure 10) [62,63]. Having been isolated from *Lippia dulcis* (Verbenaceae), which was later reclassified as *Phyla scaberrima*, (+)-hernandulcin (**31**) was found to be about 1000 times as sweet as sucrose (**5**) [62]. Moreover, the compound showed a bitter aftertaste, very low water solubility and was found to decompose upon heating [62,64]. Its natural derivative (+)-4β-hernandulcin (**31**) also showed a sweet taste and better water solubility, though a detailed characterization could not be performed due to low substance quantities [63].

Another very interesting sesquiterpenoid, mukurozioside IIb (**33**) was isolated from the fruit of *Sapindus rarak* (Sapindaceae) [63]. Evaluation of the compound’s sweetening potential revealed that its sweetness was comparable to that of sucrose (**5**) and that the effect derived from the high content (6.3%) in the fruit pulp.

With regard to receptor binding, compounds **31** and **32** are likely to bind to the same site as the monoterpenes, as both compounds are of small size and show rather pronounced lipophilicity. The structure of compound **33**, instead, indicates binding at the orthosteric site in the VFD of TAS1R2. As the linkage of sugar moieties attached reminds of other specific TAS1R2 binders, such as steviosides, it is likely that also compound **33** acts exclusively at this subunit [26].

### 3.6. Diterpenes

#### 3.6.1. Kauran-Type Diterpenoids

**Figure 11:**
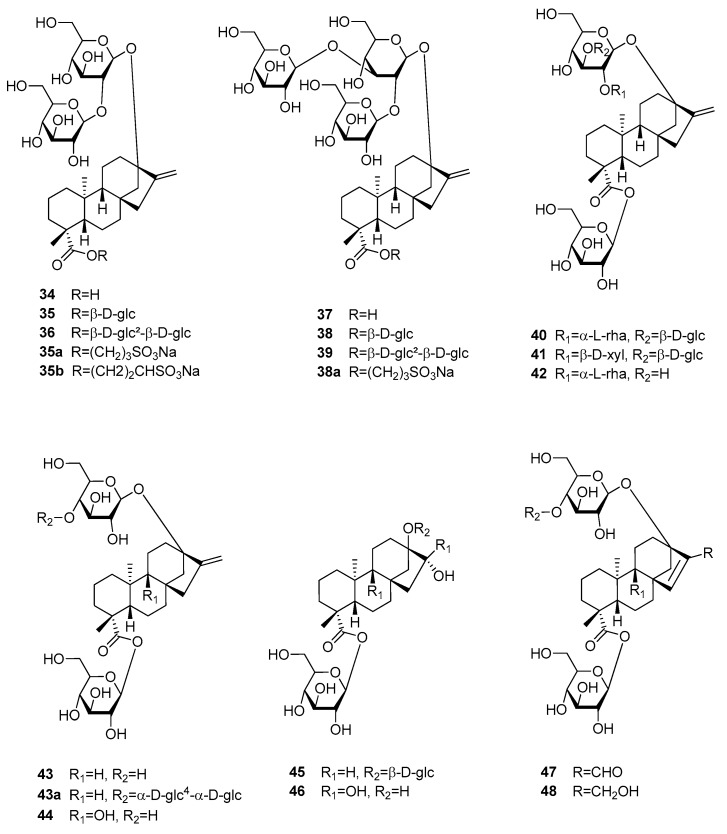
Chemical structures of sweet-tasting kauran-type diterpenoids.

Stevia glycosides belong to the most important natural sweeteners and currently are maybe the most popular plant-derived sugar substitutes. These Kauran-type diterpenoids are present in the leaves of *Stevia rebaudiana* (Asteraceae), which has been used for centuries by the indigenous population of Paraguay [12]. After stevioside (**35**) was first isolated from the leaves of “Yerba dulce” in 1931, additional glycosides were discovered since the 1970s, of which nine were studied for their sweetening properties (**34**, **36–42**) (Figure 11) [65,66,67,68]. Of the total amount of glycosides, which can vary between 4 and 20% of dried leaves, stevioside (**35**) and rebaudioside A (**38**) are present in the highest concentrations [67,69]. The latter compound has the GRAS status and is marketed as rebiana [68,70]. It is also the sweetest among the glycosides and said to have the least, though still considerable, bitter and lingering aftertaste [11]. To get rid of these drawbacks several derivatives were prepared, of which compound **35a** was still slightly bitter, while compounds **35b** and **38a** were devoid of bitterness [71]. However, their sweetness was also somewhat decreased. Regarding the sweetening potential of the natural glycosides the compounds showing branched sugar moieties (**37–39**) were sweeter than each of their non-branched counterparts (**34–36**). Within each group, the component with a monoglucosidic ester was the sweetest, which were stevioside (**35**) and rebaudioside A (**38**). Compounds containing an α-l-rhamnose moiety (**40** and **42**), showed significantly decreased RS values of 30, while substitution with a β-d-xylose moiety only slightly altered relative sweetness (**41**). The reported RS values of stevia glycosides are in line with a recent molecular docking study, which found the lowest binding energy—and thus highest sweetness—for rebaudioside A (**38**) and the highest binding energy for rebaudioside C (**40**) [72]. The authors, furthermore, suggested the rhamnose moiety as a good discriminator between bitter and sweet tastes.

Another series of kauran-type glycosides (**43–48**) was isolated from *Rubus suavissimus* (Rosaceae), which is now classified as *Rubus chingii* var. *suavissimus* [73,74]. Rubusoside (**43**) was found to have a RS of about 115 time that of sucrose (**5**), but a slightly bitter aftertaste. Semi-synthetic modification of rubusoside with a maltotriosyl moiety (**43a**) increased the RS and at the same time reduced the bitter taste [75]. Unfortunately, RS values of the other glycosides were not obtained. However, only those compounds with glucose moieties in positions 13 and 19 were found sweet, namely suaviosides B (**44**), G (**45**), I (**46**), H (**47**), and J (**48**) [74].

#### 3.6.2. Lanostan- and Gibban-Type Diterpenoids

**Figure 12:**
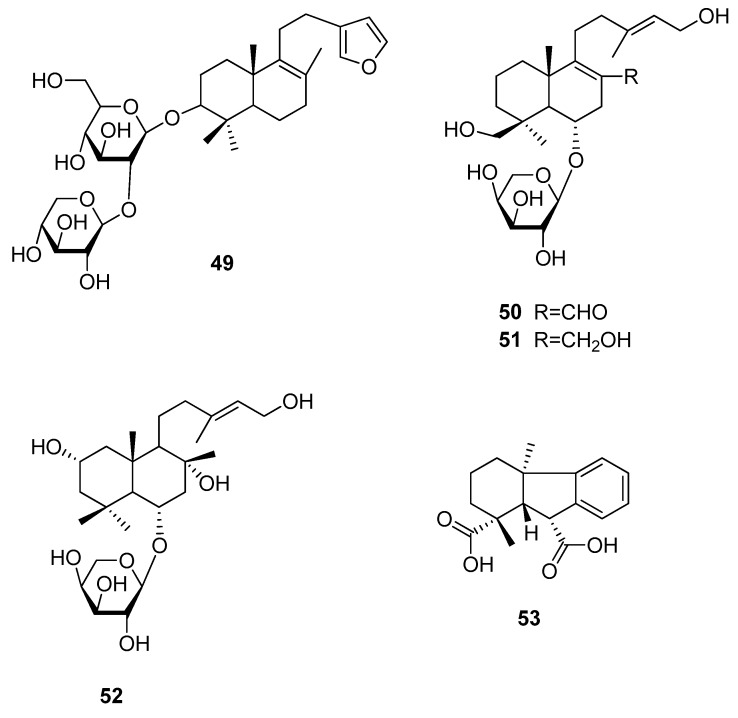
Chemical structures of sweet-tasting lanostan- and gibban-type diterpenoids.

Also, lanostan-type triterpenoids have been found to potentially exhibit a sweet taste, though not as many as reported for kauran-type diterpenoids. One of these diterpenoids, bayunoside (**49**) was found to possess a RS of about 500 times of that of glucose (Figure 12) [11]. However, the compound, which was isolated from *Salvia digitaloides* (Lamiaceae), meanwhile named *Phlomoides betonicoides*, furthermore showed a lingering aftertaste lasting for more than one hour and therefore restricting its use for many applications.

Sweet-tasting gaudiachaudiosides A (**50**), B (**51**), and E (**52**) were isolated from *Baccharis gaudichaudiana* (Asteraceae) [64]. Of these compounds, gaudichaudioside A (**50**) showed a pleasant and sweet taste that was 55 times sweeter than a 2% (*w*/*v*) sucrose solution. Gaudichaudioside B (**51**) and E (**52**), instead, showed a sweet-bitter taste, while additional compounds of this group were either neutral or entirely bitter.

The sweet gibban derivative **53** was isolated from pine tree rosin and was found to exhibit a RS of about 1600 times of that of glucose, but also a relative bitterness of 15 times of that of caffeine [76]. Because of the fact that only one of four possible isomers was found to be sweet and subsequent reports on the hormone-like effect on plant growth, the research on this compound was not further conducted [47].

### 3.7. Triterpenes

#### 3.7.1. Oleanan-Type Triterpenoids

**Figure 13:**
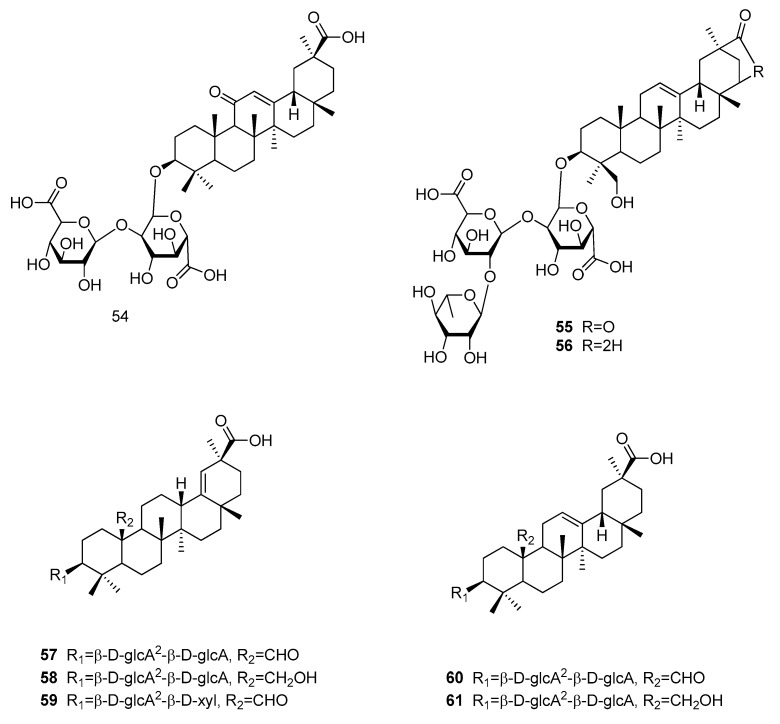
Chemical structures of sweet-tasting oleanan-type triterpenoids.

Glycyrrhzicic acid (**54**), is the sweet principle in the roots of *Glycyrrhiza glabra* (Fabaceae), where it is contained in amounts of 6–14% as calcium, magnesium and potassium salt (Figure 13) [10,12]. The mixture of the different salts is also referred to as glycyrrhizin, whereas the crude extract is known as licorice [10]. The ammonium salt of glycyrrhizic acid (**54**) hast GRAS status for the use as flavoring compound (but not as sweetener) in the US, while the Committee of Food in the EU suggests to consume not more than 100 mg per day, due to the possibility of pseudoaldosteronism [12]. The relative sweetness of glycyrrhizin compared to sucrose (**5**) is about 50, while for glycyrrhizic acid (**54**) RS values of 93 to 170 have been reported [12,77]. Apart from its sweetness, glycyrrhizic acid (**54**) is characterized by a licorice aftertaste, and shows both a slow onset as well as long sweetness lingering [10], thus making it an ideal candidate for studying receptor kinetics. Attempts to alter the compound’s taste profile by modifying the saccharide units, have, so far, been unsuccessful [78,79].

Stems of *Albizia myriophylla* (Fabaceae) are used in traditional medicine in Thailand and Vietnam and were found to contain sweet-tasting oleanan-type triterpenoids, namely albiziasaponin A and B (**55** and **56**) [80]. While albiziasaponin A (**55**), which is bearing a lactone ring, only exhibited a relative sweetness of 5 compared to sucrose (**5**), albiziasaponin B (**56**), which has a free carboxylic acid, showed an RS value of 600. Other sweet-tasting oleanan-type triterpenoids were isolated from the rhizomes of *Periandra dulcis* (Lamiaceae), which meanwhile was reclassified as *Periandra mediterranea* [64,81]. The plant, which is commonly known as Brazilian licorice afforded five sweet-tasting compounds, periandrin I to V (**57–61**). While the sweetness of periandrins I to IV was comparable to glycyrrhizic acid (**54**), periandrin V (**59**), which contains a terminal xylose moiety instead of glucuronic acid showed a RS of 200 times compared to sucrose (**5**) [82].

Comparison of the eight oleanan-type triterpenoids (**54–61**) makes clear that the carboxylic acid is crucial for the sweetening effect, as demonstrated by the low sweetness of the lactone albiziasaponin A (**55**). In contrast, the keto-group in position 11 of glycyrrhizic acid (**54**) does not seem essential as none of the periandrins (**57–61**) shows this feature, though being equally sweet. Within the group of periandrins, the one compound showing a xylose moiety (**59**) turned out to be slightly sweeter. This effect was already observed for xylose bearing rebaudioside F (**41**), which was much sweeter than its rhamnose bearing analog rebaudioside C (**40**). Another effect observed with the stevia glycosides was that branching trisaccharide moieties were sweeter than their disaccharidic analogs. If the branching sugar moiety is also the reason for the significantly higher sweetness of albiziasaponin B (**56**) cannot be concluded, because additional features distinguish this compound, such as the hydroxy-group in position 24.

#### 3.7.2. Cycloartan- and Dammaran-Type Triterpenoids

**Figure 14:**
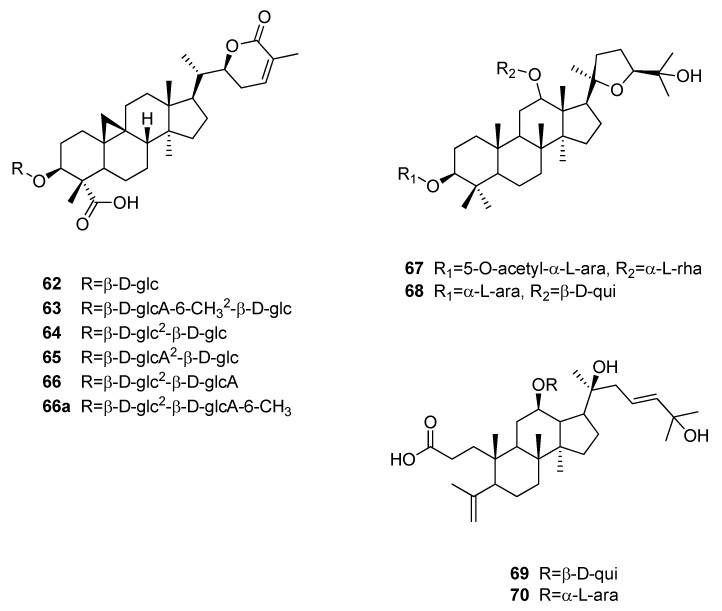
Chemical structures of sweet-tasting cycloartan- and dammaran-type triterpenoids.

Four sweet-tasting cycloartanoids, abrusosides A to D (**62**–**65**), were isolated from the leaves of *Abrus precatorius* (Fabaceae), showing RS values of 30, 100, 50, and 75, respectively (Figure 14) [83,84]. Abrusoside B (**63**), which was the sweetest of the four compounds, is bearing a glucosylated glucuronic acid methyl ester moiety. This is interesting, because abrusoside E, which was isolated in later study and showed only slight sweetness, became about 150 times sweeter than sucrose upon semi-synthetic esterification of its glucuronic acid moiety [85].

Phytochemical investigations on *Pterocarya paliurus* (Juglandaceae), which was reclassified as *Cyclocarya paliurus*, yielded dammaran-type triterpenoids cyclocaryoside A (**67**) and I (**68**) as well as 3,4-seco-dammaranes pterocaryosides A and B (**69** and **70**) [86,87,88]. While the seco-dammaranes **69** and **70** showed a relative sweetness of 50 and 100 compared to a 2% (*w*/*v*) sucrose solution, cyclocaryosides A (**67**) and I (**68**) were reported with RS values of 200 and 250, respectively. Interestingly, compounds **68** and **69** were found to contain a β-d-quinovose moiety in position C-12, which is a rarely encountered deoxyhexose in plants.

#### 3.7.3. Cucurbitane-Type Triterpenoids

**Figure 15:**
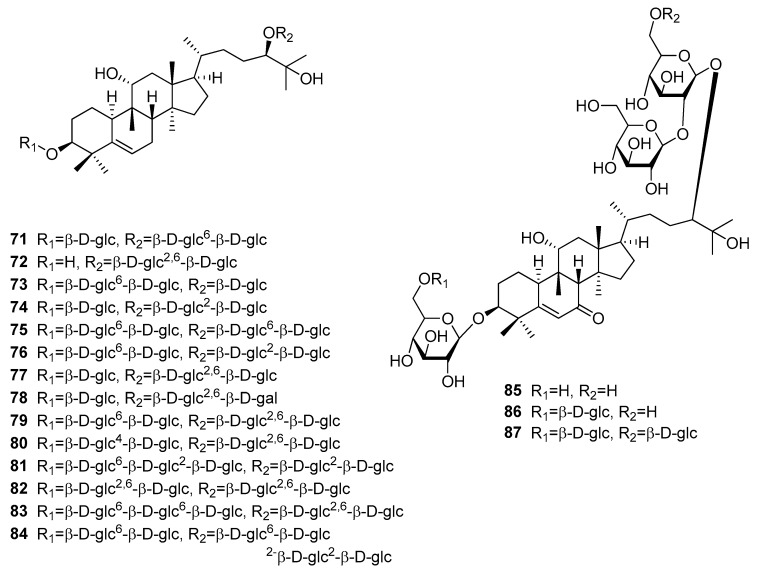
Chemical structures of sweet-tasting cucurbitan-type triterpenoids.

Mogrosides (**71–87**) belong to the most intense and most popular triterpenoid sweeteners (Figure 15) [12,68]. These compounds are the sweetening principle of *Siraitia grosvenorii* (Cucurbitaceae) fruit, also known as monk fruit or luo han guo [68]. The total amount of mogrosides in the dried fruit was determined with 3.8%, while the amount of major triterpenoid mogroside V (**79**) was found to vary between 0.5 and 1.4% [89]. Having been used for centuries in China as medicinal plant and herbal sweetener, extracts containing mogroside V (**79**) are presently used in Japan and the United States, where they are classified with the GRAS status [12,68,89]. Of the more than 50 triterpenoids isolated so far, only those bearing an α-hydroxy-group in position 11 and at least three sugar moieties exhibit a sweet taste [89,90,91,92,93]. Compounds without a hydroxy-group or with a keto-group in position 11 lose their sweetness and become bitter instead [89,91,92,93]. Though RS values are only found for the major components, at least some conclusions can be drawn. Mogroside V (**79**), which consists of five sugar moieties (a disaccharide in position C-3 and a branched trisaccharide in position C-24) has an RS value of 425 compared to a 1% (*w*/*v*) sucrose solution and is sweeter than mogrosides IVe (**76**) and VI (**82**), which consist either of two disaccharides (**76**) or two branched trisaccharides, respectively. Siamenoside I (**77**), which has only four sugar moieties has an RS value of 563 and is considered the sweetest of the mogrosides. However, the four sugar moieties of siamenoside I (**77**) are not equally divided into two disaccharides but split into a monoglucoside at position C-3 and a branched trisaccharide at position C-24. Mogroside V (**79**) was also modeled against stevioside (**35**) in a binding model of the VFD at the TAS1R2 subunit [26]. The authors found a strong linkage for mogroside V (**79**) and an additional hydrophilic interaction of the 11-hydroxy-group, which could explain the higher intensity compared to stevioside (**35**). Unfortunately, the authors confused the sugar moieties of mogroside V (**79**), and therefore no conclusions for the glycosylation pattern can be made from this study.

#### 3.7.4. Steroids

**Figure 16:**
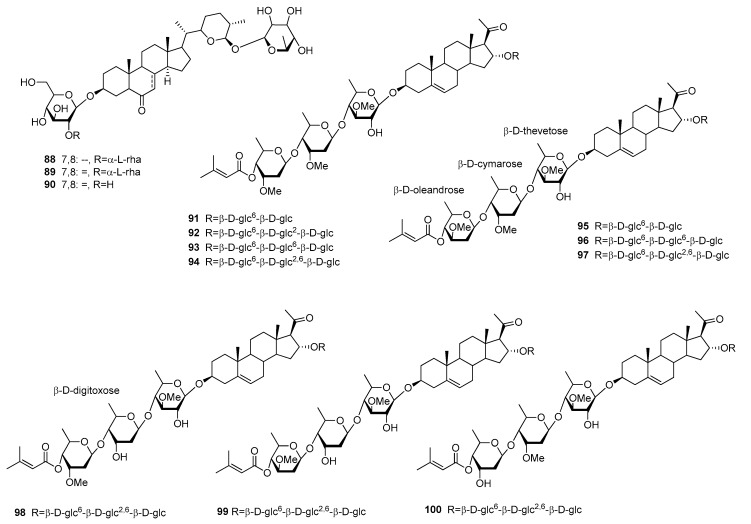
Chemical structures of sweet-tasting steroids.

The last compound class to be discussed in this review are sweet-tasting steroids (**88**–**100**) (Figure 16). Investigation of two fern species of the Polypodiaceae family, namely *Polypodium vulgare* and *Polypodium glycyrrhiza* yielded compounds osladin (**88**) as well as polypodiosides A and B (**89–90**) [94,95,96]. Osladin (**88**) and polypodioside A (**89**) were both found to be potent sweeteners, with RS values of 500 and 600, respectively. However, their poor availability coupled with low water solubility and a licorice aftertaste minimized their commercial potential [12].

Ten pregnane glycosides (**91**–**100**) were isolated from the pericarps of *Myriopteron extensum* (Apocynaceae) and named extensumsides C–L [97]. The compounds showed RS values of 50 to 400 compared to a 1% (*w*/*v*) sucrose solution and interesting substitution pattern with several methylated deoxy hexoses. These unusual sugar moieties were only linked to the hydroxy-group at position 3, whereas the saccharidic moiety at position 16 was assembled of glucose molecules. Of these moieties the highest RS values were observed for compounds with a disaccharide (**91** and **95**). Apart from that, the terminal sugar of the moiety at position 3 plays an important role, with significantly higher intensities for compounds containing β-d-cymarose instead of β-d-olandrose.

## 4. Conclusions

A total of 115 plant-derived compounds with reported sweetness (including 17 synthetic derivatives but not counting the two precursors naringin and neohesperidin) have been found in the literature and were discussed in this review. Depending on the number of similar compounds, more or fewer conclusions on their structure–activity relationships were possible. In particular, diterpenes and triterpenes were found suitable, as for these two compound (sub)classes several sweet-tasting constituents were reported. A key issue with regard to the development of new natural highly intense sweeteners is certainly the bitter aftertaste observed for many compounds. This aftertaste seems to occur independently from the respective binding site, which may be due to strong similarities with the bitter taste receptors. Though also being part of the GPCRs, the TAS2R bitter taste-receptor family encompassed around 25 members, and therefore detailed structure–activity relationships will be much more complex [98].

Still, some conclusions can be derived from the taste profiles of the already known sweeteners. One good example is the group of mogrosides, of which more than 50 members are known, both of bitter and extremely sweet tasting properties. Here, a change of the functional group at position 11 of the aglycone significantly alters the taste profile. Whereas mogrosides bearing an α-hydroxy-group are intensely sweet, compounds with a keto-function taste bitter, a fact also apparent for glycyrrhizic acid (**54**). The keto-function of the latter compound was, furthermore, found not to be relevant for its sweet flavor by comparison with other oleanan-type triterpenoids being devoid of this feature.

Moreover, an impact of the sugar moieties can be deduced from the current review. On the one hand side it seems that branching of three sugar moieties at one end of the molecule is favorable for a sweet taste, whereas at the other end a monosaccharide is superior, as observed for mogrosides as well as stevia glycosides. However, for oleanan-type triterpenoids, the distal carboxylic acid substitutes the missing second sugar moiety and was, furthermore, found crucial for the sweetening effect. Interestingly, the sweetest of the eight investigated oleanan-type triterpenoids, albiziasaponin B (**56**), was also the only one with a branched sugar moiety at position 3. However, not only does the quantity and arrangement of sugar moieties affect the amount of sweetness. Xylose moieties in many cases were found equally sweet as their glucose counterparts, whereas substitution with rhamnose in most cases led to a decrease in sweetness, as observed for dulcoside A (**42**) and rebaudioside C (**40**). Similarly, though more specifically, in the case of extensumsides a terminal cymarose moiety led to much higher sweetness than the C-3 epimeric oleandrose.

A comparison of diterpenoids with triterpenoids revealed overall higher intensities for the triterpene-type sweeteners, with some exceptions, such as e.g., bayunoside (**49**), which showed an RS value 500. A dynamical modeling study of the TAS1R2/TAS1R3 sweet taste-receptor binding either the diterpenoid stevioside (**35**) or the triterpenoid mogroside V (**79**) found that both the additional sugar moieties as well as the 11-hydroxy-group of mogroside V (**79**) caused stronger hydrogen bonds with adjacent hydrophilic residues [26]. The same results were obtained in a molecular docking study comparing mogroside V (**79**) with stevioside (**35**) and ten other terpenoid sweeteners, resulting in the lowest binding energy for mogroside V (**79**) among all investigated compounds [72].

## Figures and Tables

**Table 1:** Natural high-intensity sweeteners.

cpd.	Name	Source	RS	c (Sucrose)	Bitterness
**16**	monatin	*Schlerochiton ilicifolius*	1400	5	–
**17**	(+)-phyllodulcin	*Hydrangea macrophylla*	400	3	+
**18**	(+)-phyllodulcin 8-*O*-glucoside	*Hydrangea macrophylla*			
**19**	(+)-diyhdroquercetin 3-acetate	*Pluchea dodoneifolia*	80	2	
**20**	(+)-diyhdro-6-methoxy-kaempferol 3-acetate	*Tetraneuris turneri*	25	2	
**21**	(+)-diyhdro-6-methoxy-luetolin 3-acetate	*Tetraneuris turneri*	15	2	
**22**	(+)-diyhdro-6-methoxy-luteolin	*Tetraneuris turneri*	20	2	
**23**	glycyphyllin	*Smilax leucophylla*			++
**24**	phlorizin	*Symplocos lancifolia*			
**25**	trilobatin	*Symplocos microcalyx*			
**26**	naringin	*Citrus paradisi*			+++
**27**	neohesperidin	*Citrus x aurantium*			++
**28**	(+)-haematoxylin	*Haematoxylum campechianum*	120	3	
**29**	selligueain A	*Selliguea feei*	35	2	+
**30**	perillaldehyde	*Perilla frutescens*			
**31**	(+)-hernandulcin	*Phyla scaberrima*	1000		++
**32**	(+)-4β-hydroxyhernandulcin	*Phyla scaberrima*	n.d.		
**33**	mukurozioside IIb	*Sapindus rarak*	1		
**34**	steviolbioside	*Stevia rebaudiana*	100	10	+
**35**	stevioside	*Stevia rebaudiana*	210	0.6	+
**36**	rebaudioside E	*Stevia rebaudiana*	174		+
**37**	rebaudioside B	*Stevia rebaudiana*	150	10	+
**38**	rebaudioside A	*Stevia rebaudiana*	242		+
**39**	rebaudioside D	*Stevia rebaudiana*	221		+
**40**	rebaudioside C	*Stevia rebaudiana*	30		+
**41**	rebaudioside F	*Stevia rebaudiana*	200		+
**42**	dulcoside A	*Stevia rebaudiana*	30	30	+
**43**	rubusoside	*Rubus chingii*	114		+
**44**	suavioside B	*Rubus chingii*			
**45**	suavioside G	*Rubus chingii*			
**46**	suavioside H	*Rubus chingii*			
**47**	suavioside I	*Rubus chingii*			
**48**	suavioside J	*Rubus chingii*			
**49**	bayunoside	*Phlomoides betonicoides*	500		
**50**	gaudichaudioside A	*Baccharis gaudichaudiana*	55	2	–
**51**	gaudichaudioside B	*Baccharis gaudichaudiana*			++
**52**	gaudichaudioside E	*Baccharis gaudichaudiana*			++
**53**	4β,10α-dimethyl-1,2,3,4,5,10-hexahydrofluorene-4α,6-dicarboxylic acid	*Pinus sp.*	1600		+++
**54**	glycyrrhizic acid	*Glycyrrhiza glabra*	93–170		++
**55**	albiziasaponin A	*Albizia myriophylla*	5		
**56**	albiziasaponin B	*Albizia myriophylla*	600		
**57**	periandrin I	*Periandra mediterranea*	93–170		+
**58**	periandrin III	*Periandra mediterranea*	93–170		+
**59**	periandrin V	*Periandra mediterranea*	200		+
**60**	periandrin II	*Periandra mediterranea*	93–170		+
**61**	periandrin IV	*Periandra mediterranea*	93–170		
**62**	abrusoside A	*Abrus precatorius*	30		–
**63**	abrusoside B	*Abrus precatorius*	100		–
**64**	abrusoside C	*Abrus precatorius*	50		–
**65**	abrusoside D	*Abrus precatorius*	75		–
**66**	abrusoside E	*Abrus precatorius*			
**67**	cyclocarioside A	*Cyclocarya paliurus*	200		
**68**	cyclocarioside I	*Cyclocarya paliurus*	250		
**69**	pterocaryoside A	*Cyclocarya paliurus*	50	2	+
**70**	pterocaryoside B	*Cyclocarya paliurus*	100	2	+
**71**	mogroside III	*Siraitia grosvenorii*			–
**72**	mogroside IIIA1	*Siraitia grosvenorii*			–
**73**	mogroside IIIA2	*Siraitia grosvenorii*			–
**74**	mogroside IIIE	*Siraitia grosvenorii*			–
**75**	mogroside IVA	*Siraitia grosvenorii*			–
**76**	mogroside IVE	*Siraitia grosvenorii*	392	1	–
**77**	siamenoside I	*Siraitia grosvenorii*	563	1	–
**78**	grosmomoside	*Siraitia grosvenorii*			–
**79**	mogroside V	*Siraitia grosvenorii*	425	1	–
**80**	isomogroside V	*Siraitia grosvenorii*			–
**81**	neomogroside	*Siraitia grosvenorii*			–
**82**	mogroside VI	*Siraitia grosvenorii*			–
**83**	mogroside VIA	*Siraitia grosvenorii*			–
**84**	mogroside VIB	*Siraitia grosvenorii*			–
**85**	7-oxomogroside IIIE	*Siraitia grosvenorii*			
**86**	7-oxomogroside IV	*Siraitia grosvenorii*			
**87**	7-oxomogroside V	*Siraitia grosvenorii*			
**88**	osladin	*Polypodium vulgare*	500		+
**89**	polypodoside A	*Polypodium glycyrrhiza*	600	6	+
**90**	polypodoside B	*Polypodium glycyrrhiza*			
**91**	extensumside C	*Myriopteron extensum*	400	1	
**92**	extensumside E	*Myriopteron extensum*	200	1	
**93**	extensumside F	*Myriopteron extensum*	200	1	
**94**	extensumside H	*Myriopteron extensum*	200	1	
**95**	extensumside D	*Myriopteron extensum*	300	1	
**96**	extensumside G	*Myriopteron extensum*	100	1	
**97**	extensumside I	*Myriopteron extensum*	150	1	
**98**	extensumside J	*Myriopteron extensum*	100	1	
**99**	extensumside K	*Myriopteron extensum*	50	1	
**100**	extensumside L	*Myriopteron extensum*	50	1	

**Table 2:** Synthetic plant-derived high-intensity sweeteners.

cpd.	Name	Derived From	RS	c (Sucrose)	Bitterness
**5a**	sucralose	sucrose			
**17a**	2-(3-hydroxy-4-methoxypheyl)-1,3-benzodioxan	phyllodulcin	3000		
**17b**	2-(3-hydroxy-4-methoxypheyl)-1,4-benzodioxan	phyllodulcin	450		
**17c**	(+)-2-(3-hydroxy-4-methoxypheyl)-1,3-benzoxathian	phyllodulcin	18000		
**17d**	(+)-2-(3-hydroxy-4-methoxypheyl)-1,3-benzodithian	phyllodulcin	20000		
**19a**	4′-methyldihydro-quercetin 3-acetate	(+)-diyhdroquercetin 3-acetate	400	2	–
**26a**	naringin dihydrochalcone	naringin	300	5	
**27a**	neohesperidin dihydrochalcone	neohesperidin	1000	5	+
**27b**	3′carboxy-hesperetin	neohesperidin	3400	6	+
**28a**	(6aS,11aS)-8-methoxy-11a-methyl-6,11-dihydroindeno[1,2-c]chromene-6a,9-diol	(+)-haematoxylin	50	3	
**30a**	perillartine	perillaldehyde	370		++
**30b**	4-(methoxymethyl)-1,4-cyclohexadiene-1-carboxaldehyde *syn*-oxime	perillaldehyde	450		
**35a**	steviolbioside sulfopropyl ester sodium salt	stevioside	160		+
**35b**	steviolbioside sulfoprop-2-yl ester sodium salt	stevioside	120		–
**38a**	rebaudioside B sulfopropyl ester sodium salt	rebaudioside A	170		–
**43a**	13-*O*-β-maltotriosyl-19-*O*-β-d-glucosylsteviol	rubusoside	298		–
**66a**	abrusoside E 6′’-*O*-methyl ester	abrusoside E	150		
